# Investigating Health Disparities Associated With Multisystem Inflammatory Syndrome in Children After SARS-CoV-2 Infection

**DOI:** 10.1097/INF.0000000000003689

**Published:** 2022-09-07

**Authors:** Laura D. Zambrano, Kathleen N. Ly, Ruth Link-Gelles, Margaret M. Newhams, Manzilat Akande, Michael J. Wu, Leora R. Feldstein, Keiko M. Tarquinio, Leila C. Sahni, Becky J. Riggs, Aalok R. Singh, Julie C. Fitzgerald, Jennifer E. Schuster, John S. Giuliano, Janet A. Englund, Janet R. Hume, Mark W. Hall, Christina M. Osborne, Sule Doymaz, Courtney M. Rowan, Christopher J. Babbitt, Katharine N. Clouser, Steven M. Horwitz, Janet Chou, Manish M. Patel, Charlotte Hobbs, Adrienne G. Randolph, Angela P. Campbell

**Affiliations:** From the * COVID-19 Response Team, Centers for Disease Control and Prevention, Atlanta, Georgia; † Public Health Service Commissioned Corps, Rockville, Maryland; ‡ Department of Anesthesiology, Critical Care, and Pain Medicine, Boston Children’s Hospital, Boston, Massachusetts; § Department of Pediatrics-Section of Critical Care, The University of Oklahoma College of Medicine, Oklahoma City, Oklahoma; ¶ Division of Critical Care Medicine, Department of Pediatrics, Emory University School of Medicine, Children’s Healthcare of Atlanta, Atlanta, Georgia; ‖ Department of Pediatrics, Texas Children’s Hospital and Baylor College of Medicine, Immunization Project, Houston, Texas; ** Department of Anesthesiology and Critical Care Medicine; Division of Pediatric Anesthesiology & Critical Care Medicine, Johns Hopkins School of Medicine, Baltimore, Maryland; †† Pediatric Critical Care Division, Maria Fareri Children’s Hospital at Westchester Medical Center and New York Medical College, Valhalla, New York; ‡‡ Division of Critical Care, Department of Anesthesiology and Critical Care, University of Pennsylvania Perelman School of Medicine, Philadelphia, Pennsylvania; §§ Division of Pediatric Infectious Disease, Department of Pediatrics, Children’s Mercy Kansas City, Kansas City, Missouri; ¶¶ Department of Pediatrics, Division of Critical Care, Yale University School of Medicine, New Haven, Connecticut; ‖‖ Department of Pediatrics, School of Medicine, Seattle Children’s Research Institute, University of Washington, Seattle, Washington; *** Division of Pediatric Critical Care, University of Minnesota Masonic Children’s Hospital, Minneapolis, Minnesota; ††† Division of Critical Care Medicine, Department of Pediatrics, Nationwide Children’s Hospital, Columbus, Ohio; ‡‡‡ Department of Pediatrics, Sections of Critical Care Medicine and Infectious Diseases, University of Colorado School of Medicine and Children’s Hospital Colorado, Aurora, Colorado; §§§ Division of Pediatric Critical Care, Department of Pediatrics, SUNY Downstate Health Sciences University, Brooklyn, New York; ¶¶¶ Division of Pediatric Critical Care Medicine, Department of Pediatrics, Indiana University School of Medicine, Riley Hospital for Children, Indianapolis, Indiana; ‖‖‖ Division of Pediatric Critical Care Medicine, Miller Children’s and Women’s Hospital of Long Beach, Long Beach, California; **** Department of Pediatrics, Hackensack Meridian School of Medicine, Hackensack, New Jersey; †††† Department of Pediatrics, Division of Critical Care, Bristol-Myers Squibb Children’s Hospital, New Brunswick, New Jersey; ‡‡‡‡ Division of Immunology, Boston Children’s Hospital, Boston, Massachusetts; §§§§ Department of Pediatrics, Harvard Medical School, Boston, Massachusetts; Departments of; ¶¶¶¶ Pediatrics; ‖‖‖‖ Microbiology, Division of Infectious Diseases, University of Mississippi Medical Center, Jackson, Mississippi; ***** Department of Anesthesia, Harvard Medical School, Boston, Massachusetts.

**Keywords:** children, coronavirus disease 2019, multisystem inflammatory syndrome in children, SARS-CoV-2, health disparities, risk factors

## Abstract

Multisystem inflammatory syndrome in children (MIS-C) is a postinfectious severe acute respiratory syndrome coronavirus 2 (SARS-CoV-2)–related complication that has disproportionately affected racial/ethnic minority children. We conducted a pilot study to investigate risk factors for MIS-C aiming to understand MIS-C disparities.

**Methods:**

This case-control study included MIS-C cases and SARS-CoV-2-positive outpatient controls less than 18 years old frequency-matched 4:1 to cases by age group and site. Patients hospitalized with MIS-C were admitted between March 16 and October 2, 2020, across 17 pediatric hospitals. We evaluated race, ethnicity, social vulnerability index (SVI), insurance status, weight-for-age and underlying medical conditions as risk factors using mixed effects multivariable logistic regression.

**Results:**

We compared 241 MIS-C cases with 817 outpatient SARS-CoV-2-positive at-risk controls. Cases and controls had similar sex, age and U.S. census region distribution. MIS-C patients were more frequently previously healthy, non-Hispanic Black, residing in higher SVI areas, and in the 95th percentile or higher for weight-for-age. In the multivariable analysis, the likelihood of MIS-C was higher among non-Hispanic Black children [adjusted odds ratio (aOR): 2.07; 95% CI: 1.23–3.48]. Additionally, SVI in the 2nd and 3rd tertiles (aOR: 1.88; 95% CI: 1.18–2.97 and aOR: 2.03; 95% CI: 1.19–3.47, respectively) were independent factors along with being previously healthy (aOR: 1.64; 95% CI: 1.18–2.28).

**Conclusions:**

In this study, non-Hispanic Black children were more likely to develop MIS-C after adjustment for sociodemographic factors, underlying medical conditions, and weight-for-age. Investigation of the potential contribution of immunologic, environmental, and other factors is warranted.

## INTRODUCTION

Multisystem inflammatory syndrome in children (MIS-C) is a hyperinflammatory condition associated with immune dysregulation and multiple organ system involvement that occurs after infection with severe acute respiratory syndrome coronavirus 2 (SARS-CoV-2), the virus causing coronavirus disease 2019 (COVID-19).^1–3^ MIS-C is a distinct syndrome, although some clinical features overlap with other hyperinflammatory syndromes, such as Kawasaki disease and toxic shock syndrome,^4–7^ and with acute COVID-19 critical illness in children with multiorgan dysfunction.^8^

Immune risk factors that may predispose children to developing MIS-C after SARS-CoV-2 infection are under investigation; however, there is a consistent overrepresentation of children from minority populations relative to the estimated population distribution of reporting countries.^4,5,8–12^ A study published with data from 3 academic centers in Boston, Massachusetts, reported that higher risk for MIS-C in Black and Hispanic children remained after adjusting for the association between socioeconomic status and social vulnerability index (SVI), a measure developed by the U.S. Centers for Disease Control and Prevention (CDC).^13^ A CDC report on MIS-C national surveillance data has also noted that non-Hispanic Black children had higher odds of more severe MIS-C, with higher frequencies of intensive care unit admission and decreased cardiac function.^14^ Given the higher frequency of obesity and potential exposure to environmental contaminants in socially vulnerable communities,^15^ it is unclear if these or other factors may in part explain higher risk of MIS-C in Black children.

Understanding why health disparities by race and ethnicity are associated with MIS-C is a public health priority. We conducted this retrospective multicenter pilot study to inform a future in-depth MIS-C risk factor investigation. We included sites from the Overcoming COVID-19 multicenter U.S. network to identify MIS-C cases and at-risk SARS-CoV-2-positive outpatient controls to explore risk factors associated with MIS-C. Demographic characteristics, SVI, specific underlying medical conditions, and high weight-for-age were assessed to determine how they may independently contribute to the likelihood of MIS-C after SARS-CoV-2 infection.

## METHODS

Overcoming COVID-19 is a multicenter pediatric public health surveillance registry of hospitalized children with severe COVID-19 and MIS-C from >60 hospitals in 31 states across the USA, and MIS-C case ascertainment methods have been previously reported in detail.^8,11^ This retrospective case-control analysis was designed as a pilot to inform a future more expansive public health investigation of risk factors for MIS-C. This investigation included MIS-C patients from 17 participating hospitals, selected based on those with the highest number of MIS-C cases reported through the Overcoming COVID-19 registry as of June 24, 2020, to maximize the total number of patients available for comparison. We aimed to enroll MIS-C at-risk outpatient controls in a 4:1 ratio. All information was extracted from patient medical records using common data elements for cases and controls.

This protocol was reviewed and approved by the Boston Children’s Hospital Institutional Review Board, which served as the single institutional review board, by participating sites, and by the CDC where it was determined to meet the requirements of public health surveillance per 45 CFR §46.101(b)(4) and exempt from patient informed consent per 45 CFR §164.506(d)(2)(ii)(B). This report conforms to the Strengthening the Reporting of Observational Studies in Epidemiology reporting guidelines for case-control studies.^16^

### Study Population

Cases included children less than 18 years of age hospitalized for MIS-C. Outpatient control patients (controls) were considered eligible if they had a positive reverse transcription–polymerase chain reaction (RT-PCR) test for SARS-CoV-2 infection at an outpatient setting during a medical visit with a clinical note and were not hospitalized for any suspected SARS-CoV-2–related complications in the study period. Hospital admission or test positivity occurred for cases and controls, respectively, from March 16, 2020 to October 2, 2020, reflecting a time period before COVID-19 vaccine availability. Cases were identified retrospectively from the Overcoming COVID-19 registry if they were less than 18 years of age and otherwise met the following MIS-C clinical criteria, adapted from the CDC case definition:^8^ (1) severe disease requiring hospitalization; (2) fever ≥38°C for ≥24 hours; (3) multisystem (≥2) organ involvement (cardiac, renal, respiratory, hematologic, gastrointestinal, dermatologic or neurologic); (4) any abnormal laboratory value reflecting evidence of inflammation (elevated C-reactive protein, erythrocyte sedimentation rate, fibrinogen, procalcitonin, d-dimer, ferritin, lactic acid dehydrogenase, or interleukin 6, elevated neutrophils, reduced lymphocytes and low albumin) and (5) laboratory evidence of current or recent SARS-CoV-2 infection by RT-PCR, serology or antigen test.

Controls were matched by age group (0–4 years, 5–12 years and 13–17 years) within each site. Outpatient settings were emergency departments, outpatient clinics and telemedicine visits at sites associated with the 17 participating hospitals. Patients with positive testing who were not evaluated by a clinician were excluded.

### Exposures and Outcomes Measured

Primary exposures of interest included demographic and socioeconomic characteristics, weight-for-age as a proxy for obesity and underlying medical conditions. The primary outcome of interest was diagnosis of MIS-C.^17^ Factors considered as potential MIS-C risk factors in each model included sex, age (as a continuous variable), race, ethnicity, underlying health conditions, health insurance status and the CDC SVI (Supplemental Digital Content 2, http://links.lww.com/INF/E810), a composite index of 15 census variables broadly reflective of social conditions and community characteristics.^15,18^ Patients were considered to be previously healthy if they had no documented medical diagnoses and were not taking prescription medications for a chronic condition.

Race and ethnicity information was collected from the medical record and categorized as non-Hispanic White, non-Hispanic Black, non-Hispanic Asian, Hispanic, other, and unknown.^19^ Using established methods, persons of Hispanic ethnicity were classified as such regardless of race.^19^ SVI scores^15,18^ were linked to patients by the first four digits of their zip code (full zip code was not included in the registry to protect patient confidentiality), extrapolated to census tract using the U.S. Postal Service tract-to-zip code crosswalk file from the U.S. Department of Housing and Urban Development, and divided into tertiles (0–0.32, 0.33–0.66 and 0.67–1.00).^20^ Where a zip code matched to multiple census tracts, a weighted average for SVI was computed using tract-based population estimates derived from the American Community Survey 2014–2018 estimates,^21^ embedded within the SVI dataset.

Weight-for-age was used as a proxy to assign potential obesity status in lieu of body mass index given insufficient data; height was not available for many outpatient controls. This method also allowed inclusion of patients less than 2 years for whom obesity cannot be defined. There is, however, high concordance of weight-for-age with measures of overweight and obesity.^22^ Weight-for-age percentiles were calculated based on the 2000 CDC Growth Charts for persons from 0 to less than 20 years of age.^23,24^ Biologically implausible weight-for-age values based on extreme Z-scores of less than −5 and above 8 were excluded. Weight-for-age percentile categories included 0–89th, 90–94th and 95–100th, in accordance with established weight-for-age percentile cut-points.^24^ A sensitivity analysis was conducted excluding children under 2 years of age, for whom obesity is not defined.

### Statistical Analysis

This investigation was powered a priori to detect an OR of 2.04 given an exposure prevalence among controls of 10% and a ratio of four controls to each case, with 242 cases and 968 targeted controls using Bonferroni correction of alpha to 0.01 for multiple comparisons; however, all findings were considered exploratory, regardless of significance thresholds. Patient demographic, socioeconomic and clinical characteristics between cases and controls were compared using Kruskal-Wallis tests for nonparametric comparison of medians, Mantel-Haenszel χ^2^ tests for comparisons of frequencies, and Fisher Exact tests for comparisons of frequencies where cell sizes were less than 5. Multicollinearity of potential covariates was assessed using Pearson correlation coefficients and variance inflation factors,^25^ resulting in removal of underlying oncologic, gastrointestinal, hepatic or hematologic disease from the full model. Factors were retained in multivariable logistic regression models if their removal altered the full model effect estimate by greater than or equal to 10%. Hospital site was included as a random effect to account for between-site heterogeneity. Both SVI and weight-for-age were evaluated for their potential to modify the effect of race/ethnicity on the odds of developing MIS-C. Full models incorporated these interaction terms regardless of significance; however, race/ethnicity was stratified by weight-for-age percentile groups and by SVI tertile.

To assess the effect of missing data on the outcome, we performed multivariable multiple imputation by fully conditional specification^26^ using 20 imputed datasets as a sensitivity analysis; postimputation results are presented in the supplement. Site, race, ethnicity, weight-for-age, SVI score and insurance status were included in each imputation model. For all descriptive and modeled estimates derived from imputed data, pooled estimates were calculated into a final point estimate.

All data cleaning, management, and analysis were performed in R Studio Version 1.2.5033 and SAS version 9.4 (SAS Institute, Cary, NC).

## RESULTS

A total of 241 MIS-C cases matched to at least 1 SARS-CoV-2-positive outpatient control from 17 pediatric hospitals (Supplemental Digital Content 3 and 4, http://links.lww.com/INF/E810). After matching to cases by site and age group, 817 outpatient controls were included. All 241 patients had at least 1 positive SARS-CoV-2 test. Among them, 136 (56.4%) were positive for SARS-CoV-2 infection by RT-PCR and 194 (80.5%) were positive for SARS-CoV-2 antibody; 105 patients (43.6%) tested positive both by RT-PCR and for SARS-CoV-2 antibody. Additionally, case-patients had evidence of multisystem organ involvement (median: 4 organ systems; interquartile range: 3–5) and systemic inflammation (Supplemental Digital Content 5A and 5B, http://links.lww.com/INF/E810). All controls were positive for SARS-CoV-2 by RT-PCR and enrolled from emergency departments (60.2%), outpatient clinics (11.6%) or telemedicine with separate testing (18.7%); testing site was not reported for 9.3% of controls.

**FIGURE 1.**
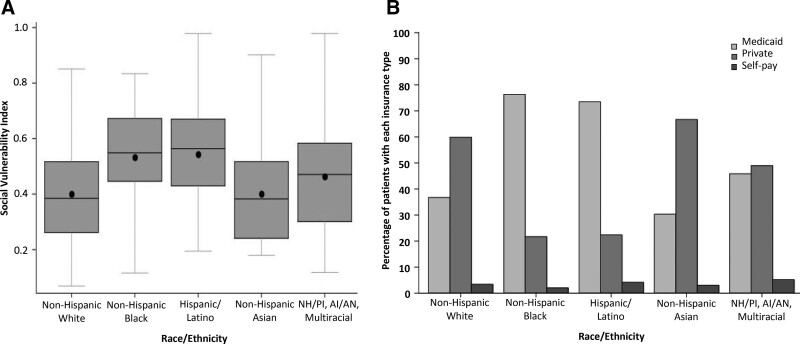
A: Distribution of social vulnerability index scores, by race and ethnicity. B: Proportion of patients with Medicaid, private insurance, or no insurance/self-pay, by race and ethnicity, among all enrolled patients.

Table 1 shows the comparison of cases to controls for demographic and socioeconomic characteristics. Sex, age and health insurance status did not significantly differ between cases and outpatient controls. A higher proportion of MIS-C cases than controls were non-Hispanic Black [88 (40.2%) vs. 226 (30.1%)], reflecting differences in the racial/ethnic distribution between groups (*P* = 0.03). Cases were observed to be more likely to reside in areas with moderate or high SVI; that is in areas with more social vulnerability. Among all enrolled patients, the distribution of SVI scores differed significantly by race (<0.001) with non-Hispanic Black and Hispanic patients having the highest SVI (Fig. 1A). More non-Hispanic White and non-Hispanic Asian patients used private insurance and more non-Hispanic Black and Hispanic patients used public insurance (Fig. 1B). Although cases were more likely to be previously healthy than the control patients, a higher proportion of cases were in the greater than or equal to 95 percentile for weight-for-age after excluding children less than 2 years of age from the analysis (Supplemental Digital Content 6, http://links.lww.com/INF/E810). Notably, while weight-for-age was used as a proxy for obesity, high concordance between weight-for-age and BMI-for-age was observed for 214 MIS-C case-patients more than 2 years of age for whom both weight and height data were available (Supplemental Digital Content 7 and 8, http://links.lww.com/INF/E810). The most common underlying respiratory condition for cases and controls was asthma.

**TABLE 1.** Distribution of Socioeconomic, Demographic, and Clinical Characteristics Among MIS-C Case-Patients and SARS-CoV-2-Positive Outpatient Controls (N = 1058)

Characteristic	Patients, No., %		*P* ^*^
	Cases (N = 241)	Controls (N = 817)	
Sex			
Female	103 (42.7)	390 (47.7)	0.17
Age			
Mean (SD)	9.0 (5.1)	8.8 (5.6)	0.51
Median (IQR)	8.7 (4.7–13.5)	9.3 (3.8–13.6)	0.51
0–4 years	62 (25.7)	232 (28.4)	0.71
5–12 years	110 (45.6)	357 (43.7)	
13–17 years	69 (28.6)	228 (27.9)	
Race and ethnicity (N = 971)^†^			
Non-Hispanic White	28 (12.8)	156 (20.7)	0.03
Non-Hispanic Black	88 (40.2)	226 (30.1)	
Non-Hispanic Asian	8 (3.7)	25 (3.3)	
NH/PI, AI/AN, non-Hispanic Multiracial	3 (1.4)	18 (2.4)	
Hispanic/Latino of any race	92 (42.0)	327 (43.5)	
Health insurance (N = 1008)^‡^			
Public (eg, Medicaid)	143 (59.3)	500 (61.2)	0.15
Private	71 (29.5)	259 (31.7)	
Uninsured/self-pay	13 (5.4)	22 (2.7)	
Census region			
Region 1: Northeast	109 (45.2)	311 (38.1)	0.25
Region 2: Midwest	37 (15.4)	150 (18.4)	
Region 3: South	70 (29.0)	260 (31.8)	
Region 4: West	25 (10.4)	96 (11.8)	
Social vulnerability index (N = 1053)^§^			
Median (IQR)	54.9 (42.7–65.6)	51.4 (35.8–62.6)	0.01
Low (Score: 0–32)	31 (12.9)	178 (21.8)	0.02
Moderate (Score: 33–66)	151 (62.7)	471 (57.6)	
High (Score: 67–100)	57 (23.7)	165 (20.2)	
Weight-for-age percentile (N = 1027)^¶^			
Median (IQR = Q3–Q1)	86.5 (55.7–97.4)	78.7 (50.4–94.7)	0.03
0–89th percentile	135 (56.0)	508 (64.6)	0.05
90th–94th percentile	31 (12.9)	89 (11.3)	
95th–100th percentile	75 (31.1)	189 (24.1)	
Respiratory system disorder	33 (13.7)	166 (20.3)	0.02
Asthma	27 (11.2)	152 (18.6)	0.01
Nonrespiratory system disorder	19 (7.9)	81 (9.9)	0.34
Cardiovascular disease	6 (2.5)	25 (3.1)	0.64
Neurologic or neuromuscular disorder	12 (5.0)	32 (3.9)	0.47
Rheumatologic or immunologic disorder	5 (2.1)	29 (3.5)	0.25
Hematologic disorder	6 (2.5)	23 (2.8)	0.79
Gastrointestinal or hepatic disorder	11 (4.6)	41 (5.0)	0.77
Endocrine disorder	6 (2.5)	16 (2.0)	0.61
Metabolic or confirmed or suspected genetic disorder	5 (2.1)	28 (3.4)	0.29
Active or prior oncologic disorder	5 (2.1)	9 (1.1)	0.10

^*^Significance was assessed using Satterthwaite *t* tests for comparison of means, Kruskal-Wallis test for Nonparametric comparison of medians, Mantel-Haenszel χ^2^ tests for comparisons of frequencies, and Fisher Exact test for comparisons of frequencies where cell sizes <5.

^†^Information on race or ethnicity was missing for 87 patients included in this analysis (22 cases and 65 controls). These missing values were excluded from the denominator.

^‡^Information on health insurance status was missing for 50 patients included in this analysis (14 cases and 36 controls).

^§^Information on zip code of residence, used to determine SVI score, was missing for 5 patients (2 cases and 3 controls).

^¶^Information on patient weight was missing for 31 controls.

AI/AN indicates American Indian/Alaskan Native; IQR, interquartile range; MIS-C, multisystem inflammatory syndrome in children; NH/PI, Native Hawaiian/Pacific Islander; SARS-CoV-2, severe acute respiratory syndrome coronavirus 2; SD, standard deviation.

In multivariable models, non-Hispanic Black children were more likely to have MIS-C [adjusted odds ratio (aOR): 2.07 (95% CI: 1.23–3.48)], as were children residing in moderate SVI areas [aOR: 1.88 (95% CI: 1.18–2.97)] and high SVI areas [aOR: 2.03 (95% CI: 1.19–3.47)] (Table 2). In general, children who were previously healthy had greater odds of MIS-C [aOR: 1.64 (95% CI: 1.18–2.28)]. While weight-for-age was not independently associated with MIS-C when including children less than 2 years of age (Table 2), after excluding these children in a sensitivity analysis, those in greater than or equal to 95th weight-for-age percentile had higher odds of developing MIS-C compared with children in the 0–89th percentile [aOR: 1.76 (1.20–2.59); Supplemental Digital Content 6, http://links.lww.com/INF/E810]. Stratified analyses indicated that among children residing in areas with low SVI, greater odds of MIS-C were observed among non-Hispanic Black children [aOR: 8.47 (95% CI: 2.24–32.03)] compared with non-Hispanic White children (Table 3). The odds of MIS-C among non-Hispanic Black children did not appear to differ by weight-for-age percentile (Table 3).

**TABLE 2.** Adjusted ORs for MIS-C by Exposure Category (N = 973)

Exposure Category	Cases	Controls	Adjusted^†^ OR (95% CI)	
	N = 225 ^*^	N = 748		
Sex				
Male	128 (56.9)	385 (51.5)	1.34 (0.98–1.82)	
Female	97 (43.1)	363 (48.5)	REF	
Race and ethnicity				
Non-Hispanic Black	81 (36.0)	203 (27.1)	2.07 (1.23–3.48)	
Non-Hispanic Asian	8 (3.6)	25 (3.3)	1.72 (0.69–4.27)	
NH/PI, AI/AN, non-Hispanic Multiracial	23 (10.2)	69 (9.2)	1.67 (0.89–3.17)	
Hispanic/Latino of any race	85 (37.8)	310 (41.4)	1.26 (0.76–2.11)	
Non-Hispanic White	28 (12.4)	141 (18.9)	REF	
Health insurance				
U.S. Government (eg, Medicaid)	141 (62.7)	480 (64.2)	0.91 (0.64–1.30)	
Uninsured/self-pay	13 (5.8)	20 (2.7)	1.95 (0.91–4.19)	
Private	71 (31.6)	248 (33.2)	REF	
Social vulnerability index				
High (Score: 67–100)	53 (23.6)	145 (19.4)	2.03 (1.19 -3.47)	
Moderate (Score: 33–66)	143 (63.6)	438 (58.6)	1.88 (1.18–2.97)	
Low (Score: 0–32)	29 (12.9)	165 (22.1)	REF	
Weight-for-age percentile				
95th–100th percentile	68 (30.2)	180 (24.1)	1.38 (0.97–1.96)	
90th–94th percentile	29 (12.9)	85 (11.4)	1.32 (0.82–2.13)	
0–89th percentile	128 (56.9)	483 (64.6)	REF	
Underlying medical conditions^‡^				
Respiratory system disorder	29 (12.9)	154 (20.6)	0.50 (0.32–0.77)	
Nonrespiratory system disorder	19 (8.4)	76 (10.2)	1.22 (0.71–2.10)	
Previously healthy	148 (65.8)	418 (55.9)	1.64 (1.18–2.28)	

^*^Complete-case analyses included 225 cases and 748 outpatient controls, for whom data were available on all model covariates. Supplemental documentation includes models with imputed values to include all 241 cases and 817 outpatient controls included in this investigation.

^†^Adjusted models incorporated a combination of the following factors, given covariates that were confounders for each exposure of interest: Sex, age (continuous, in years), race/ethnicity, continuous SVI score, weight-for-age percentile, insurance status and the presence of underlying respiratory disorders.

^‡^For each binomial underlying medical condition variable (respiratory system disorder, nonrespiratory system disorder, or previously healthy), the reference group was considered to be those for whom the condition was absent.

AIAN indicates American Indian or Alaskan Native; MIS-C, multisystem inflammatory syndrome in children; NHPI, Native Hawaiian or Pacific Islander.

**TABLE 3.** Association Between Race/Ethnicity and MIS-C, Stratified by SVI Quartile and Weight-for-Age Percentiles (N = 1058)^*^

Characteristic	Cases	Controls	Adjusted OR (95% CI)	
Low SVI (Score: 0 to 0.32)				
Non-Hispanic Black	13 (52.0)	22 (17.5)	8.47 (2.24–32.03)	
Hispanic/Latino of any race	4 (16.0)	43 (34.1)	0.79 (0.21–2.96)	
Non-Hispanic White	8 (32.0)	61 (48.4)	REF	
Moderate to high SVI (Score: 0.33–1.0)				
Non-Hispanic Black	68 (40.2)	181 (34.3)	1.76 (0.97–3.20)	
Hispanic/Latino of any race	81 (47.9)	267 (50.6)	1.49 (0.84–2.65)	
Non-Hispanic White	20 (11.8)	80 (15.2)	REF	
0–89th WAPCT				
Non-Hispanic Black	43 (41.0)	124 (30.0)	1.72 (0.87–3.40)	
Hispanic/Latino of any race	45 (42.9)	188 (45.4)	1.13 (0.58–2.20)	
Non-Hispanic White	17 (16.2)	102 (24.6)	REF	
≥90th WAPCT				
Non-Hispanic Black	38 (42.7)	79 (32.9)	2.36 (0.96–5.82)	
Hispanic/Latino of any race	40 (44.9)	122 (50.8)	1.25 (0.53–2.95)	
Non-Hispanic White	11 (12.4)	39 (16.3)	REF	

^*^Children who were Native Hawaiian, Pacific Islander, Asian, American Indian, Alaskan Native, multiracial or other race were excluded from these stratified analyses due to insufficient sample size. The complete-case multivariable SVI- and WAPCT-stratified analyses included 194 cases and 654 outpatient controls who were either non-Hispanic Black, non-Hispanic White or Hispanic/Latino of any race.

MIS-C indicates multisystem inflammatory syndrome in children; SVI, social vulnerability index; WAPCT, weight-for-age percentile.

There were 22 (9.1%) cases and 65 (8.0%) outpatient controls missing both race and ethnicity. The distribution of demographic and socioeconomic characteristics among cases and controls after imputation of missing data are shown in Supplemental Digital Content 9, http://links.lww.com/INF/E810. After imputing missing data, our findings were similar (Supplemental Digital Content 9–11, http://links.lww.com/INF/E810).

## DISCUSSION

In this multicenter pilot case-control investigation, MIS-C was more likely among non-Hispanic Black children infected with SARS-CoV-2 than non-Hispanic White children after controlling for SVI, weight-for-age percentile, and health insurance status. Social vulnerability was highest in non-Hispanic Black and Hispanic children, but it was independently associated with MIS-C after adjusting for demographic factors and presence of underlying conditions. Over 80% of MIS-C patients were previously healthy, so underlying health conditions were not a risk factor for MIS-C. While non-Hispanic Black children are at disproportionately higher risk of SARS-CoV-2 infection,^27–29^ our analysis suggests these children were also more likely to develop MIS-C after infection, as we compared MIS-C cases to an age-matched control group of SARS-CoV-2-positive children potentially at risk for MIS-C. While Hispanic children did not have increased odds of MIS-C compared with outpatient controls with SARS-CoV-2 infection, they were highly represented in both study arms. This analysis adds to the growing body of evidence demonstrating the disproportionate impact of COVID-19 on minority communities.^30–33^ A combination of unmeasured factors and social determinants, such as household and localized environmental characteristics, diet and general nutrition, host factors that alter immune cell recognition of SARS-CoV-2,^4^ and chronic stress-induced hyperinflammation, may play a large contributory role in the susceptibility to and pathophysiology of MIS-C.^34^

In contrast to a prior case-control study,^13^ we cannot attribute the higher frequency of MIS-C in non-Hispanic Black children to increased risk of SARS-CoV-2 infection. As race and ethnicity are social constructs, we attempted to use SVI to interpret the associations within the broader context of social inequities and related social determinants of health.^35^ In the US, census tract of residence may explain 70% of variation in individual health outcomes,^36^ and factors associated with a person’s residence and socioeconomic status have been identified as independent risk factors for outcomes related to SARS-CoV-2 infection.^13,36^ Our study is consistent with the findings of Javalkar et al^13^ showing Black race and higher SVI to be independently associated with MIS-C. Our stratified analysis, however, further demonstrated that SVI modified the association between race and MIS-C such that non-Hispanic Black children who resided in neighborhoods with lower SVI were more likely to develop MIS-C compared with non-Hispanic White children; however, this association was attenuated among children residing in neighborhoods with moderate to high SVI. Our finding that SVI modified the association between race and MIS-C suggests the importance of socioeconomic stress to the dysregulated immune response characteristic of MIS-C, also seen in murine and nonhuman primate models of social stress associated with increased immune-mediated inflammation.^37,38^ It is also possible that aggregate neighborhood measures of social factors may not fully capture the heterogeneity of individual and/or household-level social risk factors within a neighborhood. SVI may not include the specific factors, such as household crowding, that may explain the racial differences in risk for MIS-C among children who reside in areas with lower SVI. Understanding community drivers of racial disparities can promote equitable distribution of public health interventions, including COVID-19 vaccination, which is likely protective against MIS-C but has noted disparities in access and uptake by race and ethnicity.^39,40^ These interventions must be tailored to specific neighborhood-level risk factors and encourage a public health and systems approach to reducing inequities and fostering community health.^41^

This exploratory pilot investigation is subject to several limitations. Control patients presenting to outpatient facilities may be more likely to have symptomatic COVID-19 triggering the testing, whereas many children developing MIS-C tend to have asymptomatic or mild primary infection.^42,43^ Underlying respiratory diseases, like asthma, are associated with symptomatic COVID-19;^44^ therefore, outpatient visits associated with acute infection may have been more common for this outpatient control population compared with MIS-C patients.^45,46^ Second, controls may have more frequent engagement with preventive care services, which may be linked to both higher burden of underlying disease and lower social vulnerability. Third, MIS-C patients seeking care at specialized pediatric hospitals may be drawn from a broader geographic catchment area compared with outpatients, and we did not match by zip code. Fourth, data collection for this investigation relied solely on data available in medical records with missing data for some variables; however, imputed and observed data were comparable. Available data also did not leverage standardized MIS-C adjudication criteria at the point of data collection, as this project was conducted during the first year of the pandemic, concurrent with criteria development and implementation for the Overcoming COVID-19 registry. Relatedly, alternative diagnoses caused by coinfections cannot be ruled out, as 87 MIS-C patients in this study were not screened for other pathogens, and cultures were less routinely ordered for early MIS-C cases. However, all patient data subsequently underwent independent adjudication by CDC coinvestigators, and only patients meeting CDC’s clinical criteria for MIS-C were included as case-patients. Fifth, studies in the US repeatedly show that while reporting of White race is accurate, non-White race is less accurately reflected when using electronic medical records.^47–53^ While race and ethnicity data were obtained from the medical record, it is unknown whether this information was self-indicated by the patient or recorded by providers. Sixth, we used census tracts extrapolated from zip-code SVI data to define subjects’ neighborhoods, which has limitations but has been used in prior studies.^54–56^ Finally, during enrollment, it was not possible to frequency match four outpatient controls to each case-patient by age group and site; however, we incorporated age as a continuous variable in all adjusted models.

## CONCLUSIONS

This investigation provides further evidence that non-Hispanic Black children appear to have higher likelihood of developing MIS-C after SARS-CoV-2 infection after adjustment for high weight-for-age as a proxy for obesity and social vulnerability measures. Most children with MIS-C are previously healthy so underlying health conditions are unlikely to be major risk factors. Investigations of other households, environmental and immunologic triggers for MIS-C are warranted to identify modifiable factors for MIS-C prevention.

## ACKNOWLEDGMENTS

Overcoming COVID-19 Investigators: See Supplemental Digital Content 1, http://links.lww.com/INF/E810, for list of collaborators.

## Supplementary Material


