# Supplementary material for: Investigating Health Disparities Associated With Multisystem Inflammatory Syndrome in Children After SARS-CoV-2 Infection
Source: Pediatr Infect Dis J. 2022 Sep 7;41(11):891–8. doi: 10.1097/INF.0000000000003689 (PMC9555608; doi:10.1097/INF.0000000000003689)
Supplement: Supplementary file 1 [file inf-41-891-s001.docx]

**SUPPLEMENTARY DIGITAL CONTENT**

**Supplemental Digital Content 1. Overcoming COVID-19 Investigators**

**(Listed in PubMed, and ordered by U.S. State)**

The following study group members were all closely involved with the design, implementation, and oversight of the Overcoming COVID-19 study.

**California:** Miller Children’s & Women’s Hospital Long Beach, Long Beach. Christopher J. Babbitt, MD.

**Colorado:** Children’s Hospital Colorado, Aurora. Aline B. Maddux, MD, MSCS ; Christina M. Osborne, MD., Sara Shankman, DNP, CPNC-AC

**Connecticut:** Yale New-Haven Children’s Hospital, New Haven. John S. Giuliano, Jr., MD.

**Georgia:** Children's Healthcare of Atlanta at Egleston, Atlanta. Keiko M. Tarquinio, MD.

**Indiana:** Riley Hospital for Children, Indianapolis. Courtney M. Rowan, MD, MS.

**Maryland:** Johns Hopkins Children’s Hospital, Baltimore. Becky J. Riggs, MD; Melania M. Bembea, MD, MPH, PhD.

**Massachusetts:** Boston Children’s Hospital, Boston. Adrienne G. Randolph, MD; Margaret M. Newhams, MPH; Sabrina R. Chen; Cameron C. Young.

**Minnesota:** University of Minnesota Masonic Children’s Hospital, Minneapolis, Janet R. Hume, MD, PhD.

**Missouri:** Children’s Mercy Hospital, Kansas City. Jennifer E. Schuster, MD.

**New Jersey:** Hackensack University Medical Center, Hackensack. Katharine N. Clouser, MD.

**New Jersey:** Bristol-Myers Squibb Children's Hospital, New Brunswick. Lawrence C. Kleinman, MD, MPH, FAAP; Simon Li, MD, MPH; Steven M. Horwitz, MD.

**New York:** Maria Fareri Children's Hospital, Valhalla**.** Aalok R. Singh, MD.

**New York:** SUNY Downstate Medical Center University Hospital, Brooklyn**.** Sule Doymaz, MD.

**Ohio:** Nationwide Children’s Hospital, Columbus. Mark W. Hall MD, FCCM.

**Pennsylvania:** Children’s Hospital of Philadelphia, Philadelphia. Julie C. Fitzgerald, MD, PhD, MSCE.

**Texas:** Texas Children’s Hospital and Baylor College of Medicine, Houston. Laura L. Loftis, MD; Leila C. Sahni, PhD, MPH.

**Washington:** Seattle Children’s Hospital, Seattle. Janet A. Englund, MD; John K. McGuire, MD; Lincoln S. Smith, MD.

**CDC COVID-19 Response Team on Overcoming COVID-19:** Laura D. Zambrano, PhD, MPH; Angela P. Campbell, MD, MPH; Kathleen N. Ly, MPH; Ruth Link-Gelles, PhD, MPH; Michael Wu, MSc; Leora Feldstein, PhD, MPH; Manish M. Patel, MD, MPH; Ashley M. Price, MPH; Anna Bowen, MD, MPH; Paul A. Gastañaduy, MD, MPH

**Supplemental Digital Content 2: CDC’s Social Vulnerability Index**

CDC and the Agency for Toxic Substances and Disease Registry developed the social vulnerability index (SVI) to identify particular geographic regions that are particularly vulnerable to natural disasters and infectious disease outbreaks due to socioeconomic status, household composition, minority status, or housing type and transportation. More information on the CDC/ATSDR SVI can be found at: [CDC/ATSDR Social Vulnerability Index (SVI)](https://www.atsdr.cdc.gov/placeandhealth/svi/index.html). Broadly, the SVI is a composite score of the following domains, with scores available at either the census tract- or county-level:


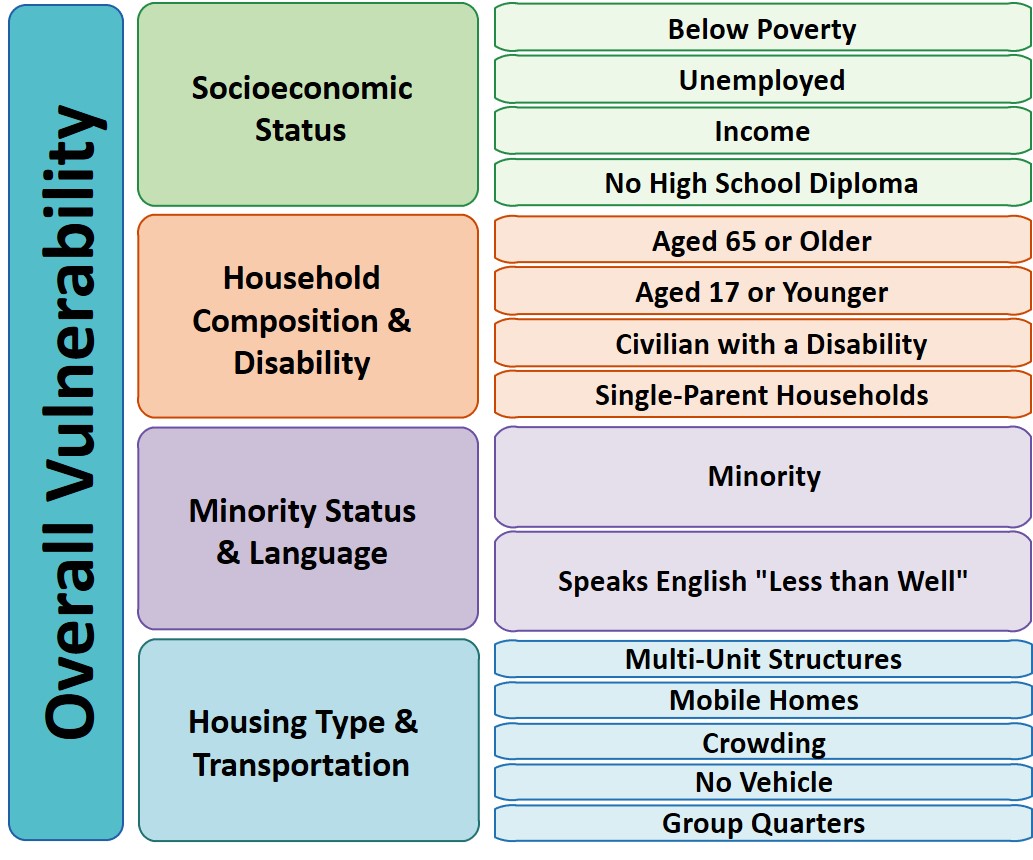


Reference: CDC SVI Documentation 2018 (Place and Health): <https://www.atsdr.cdc.gov/placeandhealth/svi/documentation/SVI_documentation_2018.html>

**Supplemental Digital Content 3.** This case-control investigation included 17 pediatric hospitals in 14 states across the United States.


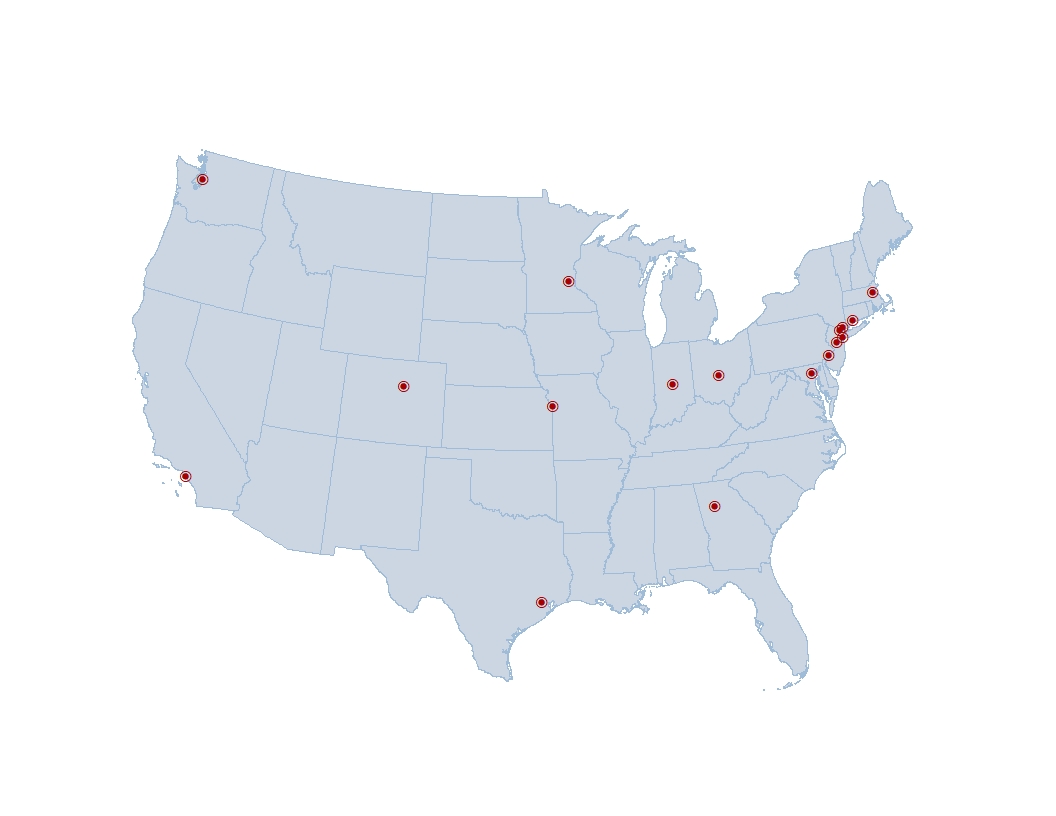


**2,012 Children hospitalized with a COVID-19-related condition in *Overcoming COVID-19* registry through December 31, 2020.**

**941 Children meeting clinical criteria for MIS-C.**

**662 Patients with hospital admission dates between March 16 and October 2, 2020.**

**642 Patients under 18 years of age.**

**322 Patients treated in one of 17 participating hospitals.**

**241 Frequency-matched with controls by site and age bracket.**

**1,071 Children hospitalized with severe acute COVID-19, but did not meet MIS-C clinical criteria.**

**279 MIS-C patients hospitalized after October 2, 2020**

**20 MIS-C patients between the ages of 18 and 20 years**

**81 MIS-C patients with no available matched control**

**Supplemental Digital Content 4.** Case-patient enrollment flow diagram from 2020 Overcoming COVID-19 pediatric patient registry.

**320 MIS-C patients treated outside of the 17 participating hospitals.**

**Supplemental Digital Content 5A.** Evidence of severe organ involvement among MIS-C case-patients.

| **Clinical characteristics** | **N (%)** |
| --- | --- |
| Number of organ systems involved (Median, IQR) | 4 (3 – 5) |
| Cardiac | 139 (57.7) |
| Respiratory | 200 (83.0) |
| Gastrointestinal | 220 (91.3) |
| Hematologic | 185 (76.8) |
| Mucocutaneous/dermatologic | 172 (71.4) |
| Neurologic | 32 (13.3) |
| Renal | 8 (3.3) |

**Supplemental Digital Content 5B.** Inflammatory marker among MIS-C case-patients.

| **Inflammatory markers** | **MIS-C (N=241)** |
| --- | --- |
| ***Maximum value during hospitalization*** |  |
| C-reactive protein (mg/dL) |  |
| No. | 235 |
| Median (IQR) | 23.4 (13.2 – 39.5) |
| Erythrocyte sedimentation rate (mm/hr) |  |
| No. | 145 |
| Median (IQR) | 63 (40 – 92) |
| Fibrinogen (mg/dL) |  |
| No. | 221 |
| Median (IQR) | 535 (427 – 638) |
| Procalcitonin (ng/mL) |  |
| No. | 148 |
| Median (IQR) | 7.31 (1.56 – 18.35) |
| D-dimer (ng/mL) |  |
| No. | 178 |
| Median (IQR) | 3535 (2180 – 7930) |
| Ferritin (ng/mL) |  |
| No. | 221 |
| Median (IQR) | 573.3 (289.0 – 1162.5) |
| Interleukin 6 (pg/mL) |  |
| No. | 84 |
| Median (IQR) | 44.0 (15.7 – 132.3) |
| Neutrophil-to-lymphocyte ratio |  |
| No. | 237 |
| Median (IQR) | 9.14 (4.83 – 18.96) |
| ***Minimum value during hospitalization*** |  |
| Albumin (g/dL) |  |
| No. | 234 |
| Median (IQR) | 2.60 (2.10 – 3.02) |

**Supplemental Digital Content 6.** Complete-case analyses of the relative likelihood for MIS-C among children in the 90^th^ - <95^th^ and 95^th^ or higher percentiles for weight-for-age, after excluding children <2 years.

|  | **No. (%)** | | |
| --- | --- | --- | --- |
| **Weight-for-age percentile** | **Case-patients (N=241)** | **Controls**  **(N=791)^b^** | **Adjusted OR^a^ (95% CI)** |
| 95-100th percentile | 71 (29.5) | 149 (18.8) | 1.76 (1.20 – 2.59) |
| 90-94th percentile | 29 (12.0) | 77 (9.8) | 1.42 (0.85 – 2.35) |
| 0-89th percentile | 115 (47.7) | 403 (51.0) | REF |
| <2 years of age | 26 (10.8) | 162 (20.5) | 0.37 (0.21 – 0.67) |

^a^ Model adjusted for sex, age (continuous, in years), race/ethnicity, insurance status, social vulnerability index score, and history of underlying respiratory disease.

^b^ Weight-for-age percentile missing for 26 of 655 (4.0%) controls aged 2 years or older. All calculations use 629 controls aged 2 years or older for whom weight-for-age information is known.

**Supplemental Digital Content 7.** Relationship between weight-for-age percentiles and BMI-for age among MIS-C case-patients. Analyses were restricted to MIS-C patients ≥2 years of age with both height and weight information available (N=214).

|  | **BMI-for-age ≥90^th^ percentile** | **BMI-for-age <90^th^ percentile** | **Total** |
| --- | --- | --- | --- |
| **Weight-for-age ≥90^th^ percentile** | 87 | 13 | 100 |
| **Weight-for-age <90^th^ percentile** | 18 | 96 | 114 |
| **Total** | 105 | 109 | 214 |

**Performance of weight-for-age percentiles as a proxy for BMI-for age:**

Sensitivity: 87/(87+18) = 82.9%

Specificity: 96/(96+13) = 88.1%

PPV: 87/(87+13) = 87.0%

NPV: 96/(96+18) = 84.2%

**Supplemental Digital Content 8.** a) Receiver operating characteristic curve, demonstrating the predictive value of weight-for-age among patients with BMI in the 90^th^ percentile or higher (AUC=0.9225). b) Correlation between continuous values of weight-for-age and BMI-for-age (ρ=0.6886, p<0.001). Analyses were restricted to MIS-C patients ≥2 years of age with both height and weight information available (N=214).


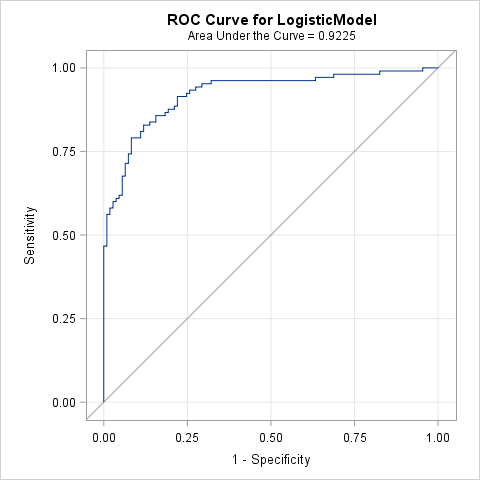


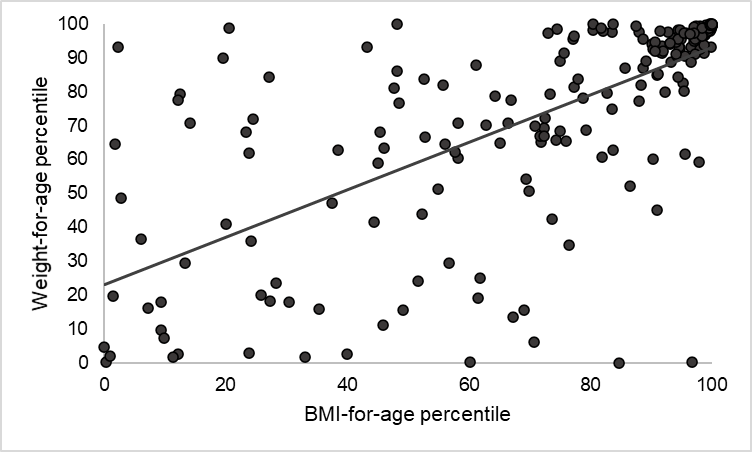


ρ=0.6886
p<0.001

**Supplemental Digital Content 9.** Distribution of race and ethnicity, health insurance status, SVI score, and weight-for-age percentiles among MIS-C case patients and outpatient controls with SARS-CoV-2 infection, after multiple imputation (N=1,058).

| **Characteristic** | **Patients, N (%)** | | | |
| --- | --- | --- | --- | --- |
|  | **Case-patients (N=241)** | **Controls (N=817)** | ***P*-value^c^** |  |
| **Race and ethnicity^a^** |  |  |  |  |
| Non-Hispanic White | 31 (13.1) | 172 (21.1) | 0.02 |  |
| Non-Hispanic Black | 98 (40.8) | 251 (30.7) |  |  |
| Non-Hispanic Asian | 9 (3.6) | 28 (3.5) |  |  |
| NHPI, AIAN, Non-Hispanic Multiracial | 4 (1.6) | 19 (2.3) |  |  |
| Hispanic/Latino of any race | 99 (40.9) | 347 (42.5) |  |  |
| **Health insurance** |  |  |  |  |
| U.S. Government (e.g., Medicaid) | 153 (63.3) | 521 (63.7) | 0.11 |  |
| Private | 75 (31.3) | 274 (33.6) |  |  |
| Uninsured / Self-pay | 13 (5.4) | 22 (2.7) |  |  |
| **Social vulnerability index (n=1,058)** |  |  |  |  |
| Median (IQR) | 54.9 (42.7 - 65.6) | 51.4 (35.8 - 62.6) | 0.01 |  |
| Low (Score: 0–32) | 31 (12.9) | 178 (21.8) | 0.01 |  |
| Moderate (Score: 33–66) | 152 (62.9) | 473 (57.9) |  |  |
| High (Score: 67 - 100) | 58 (24.2) | 166 (20.3) |  |  |
| **Weight-for-age percentile^a^** |  |  |  |  |
| Median (IQR) | 83.6 (50.2 – 97.2) | 77.1 (49.3 – 94.5) | <0.001 |  |
| 0–89th percentile | 135 (56.0) | 533 (65.2) | 0.03 |  |
| 90–94th percentile | 31 (12.9) | 91 (11.1) |  |  |
| 95–100th percentile | 75 (31.1) | 193 (23.7) |  |  |
| **Weight-for-age percentile (excluding children <2 years of age)** |  |  |  |  |
| 0-89th percentile | 115 (47.7) | 424 (51.9) | <0.001 |  |
| 90-94th percentile | 29 (12.0) | 78 (9.6) |  |  |
| 95-100th percentile | 71 (29.5) | 153 (18.7) |  |  |
| <2 years of age | 26 (10.8) | 162 (19.8) |  |  |

**Supplemental Digital Content 10.** Adjusted ORs for MIS-C by Exposure Category, using data after multiple imputation.

| **Exposure category** |  |
| --- | --- |
|  | **Adjusted^a^ OR (95% CI)** |
| **Sex** |  |
| Male | 1.11 (0.77 – 1.60) |
| Female |  |
| **Race and ethnicity** |  |
| Non-Hispanic Black | 2.17 (1.30 – 3.61) |
| Non-Hispanic Asian | 1.76 (0.71 – 4.33) |
| NH/PI, AI/AN, and Non-Hispanic Multiracial | 1.17 (0.32 – 4.20) |
| Hispanic/Latino of any race (except NH/PI or AI/AN) | 1.43 (0.86 – 2.36) |
| Non-Hispanic White | REF |
| **Health insurance** |  |
| U.S. Government (e.g., Medicaid) | 0.90 (0.64 – 1.28) |
| Uninsured / Self-pay | 1.82 (0.85 – 3.87) |
| Private | REF |
| **Social vulnerability index** |  |
| High (Score: 67 - 100) | 1.86 (1.11 – 3.10) |
| Moderate (Score: 33–66) | 1.79 (1.15 – 2.79) |
| Low (Score: 0–32) | REF |
| **Weight-for-age percentile** |  |
| 95-100th percentile | 1.48 (1.05 – 2.07) |
| 90-94th percentile | 1.37 (0.86 – 2.17) |
| 0-89th percentile | REF |
| **Weight-for-age percentile (aged 2 years or older)** |  |
| 95-100th percentile | 1.94 (1.24 – 3.03) |
| 90-94th percentile | 1.17 (0.61 – 2.25) |
| 0-89th percentile | REF |
| <2 years of age | 0.59 (0.29 – 1.18) |
| **Underlying medical conditions** |  |
| Respiratory system disorder | 0.51 (0.34 – 0.79) |
| Non-respiratory system disorderc | 0.69 (0.40 – 1.19) |
| Previously healthy | 1.60 (1.16 – 2.20) |

Abbreviations: NH/PI="Native Hawaiian or Pacific Islander"; AI/AN="American Indian or Alaskan Native"

^a^ Adjusted models incorporated a combination of the following factors, given covariates that were confounders for each exposure of interest: Sex, age (continuous, in years), race/ethnicity, continuous SVI score, weight-for-age percentile groupings, insurance status, and the presence of underlying respiratory disorders.

**Supplemental Digital Content 11.** Association between race/ethnicity and MIS-C, stratified by SVI quartile and weight-for-age percentiles (N=1,058).^a^ after multiple imputation.

|  |  | |  |
| --- | --- | --- | --- |
| **Characteristic** | **Case-patients** | **Controls** | **Adjusted OR (95% CI)** |
| **Low SVI (Score: 0 to 0.32)** |  |  |  |
| Non-Hispanic Black | 15 (48.4) | 36 (20.2) | 4.58 (1.58 - 13.32) |
| Hispanic/Latino of any race | 5 (16.1) | 49 (27.5) | 0.81 (0.43 – 2.81) |
| Non-Hispanic White | 9 (29.0) | 74 (41.6) | REF |
| **Moderate to High SVI (Score: 0.33 to 1.00)** |  |  |  |
| Non-Hispanic Black | 54 (35.5) | 157 (33.2) | 1.98 (1.10 - 3.57) |
| Hispanic/Latino of any race | 67 (44.1) | 210 (44.4) | 1.47 (0.83 - 2.61) |
| Non-Hispanic White | 20 (13.2) | 85 (18.0) |  |
| **0 - 89th WAPCT** |  |  |  |
| Non-Hispanic Black | 47 (40.9) | 130 (30.7) | 1.89 (0.99 – 3.61) |
| Hispanic/Latino of any race | 42 (36.5) | 172 (40.6) | 1.36 (0.72 – 2.59) |
| Non-Hispanic White | 18 (15.7) | 94 (22.2) | REF |
| **≥90th WAPCT** |  |  |  |
| Non-Hispanic Black | 45 (45.0) | 74 (32.0) | 2.67 (1.10 – 6.47) |
| Hispanic/Latino of any race | 42 (42.0) | 105 (45.5) | 1.48 (0.63 – 3.48) |
| Non-Hispanic White | 11 (11.0) | 44 (19.0) | REF |

Abbreviations: SVI="Social vulnerability index"; WAPCT="Weight-for-age percentile"

^a^ Imputation results that generated number of patients were rounded to the nearest whole number.
